# Identifying the thresholds of C-reactive protein, procalcitonin, and interleukin-6 among children ≤36 months’ old with fever without source at risk of serious bacterial infections: a systematic review and meta-analysis

**DOI:** 10.3389/fped.2026.1697210

**Published:** 2026-02-26

**Authors:** Natalia Sutiman, Jiaying Lin, Rehena Sultana, Sarah Hui Wen Yao, Sharon Si Min Goh, Suzanne-Kae Rocknathan, Sashikumar Ganapathy, Shu-Ling Chong

**Affiliations:** 1 Department of Emergency Medicine, KK Women’s and Children’s Hospital, Singapore, Singapore; 2 Department of Paediatric Medicine, KK Women’s and Children’s Hospital, Singapore, Singapore; 3 Centre of Quantitative Medicine, SingHealth Duke-NUS Academic Medical Centre, Singapore, Singapore; 4 Duke-NUS Medical School, Singapore, Singapore; 5 Paediatric Academic Clinical Programme, SingHealth Duke-NUS, Singapore, Singapore

**Keywords:** bacterial, biomarker, children, infection, threshold

## Abstract

**Introduction:**

Management of children ≤36 months of age presenting with fever without source remains a challenge because the underlying aetiologies may range from self-limiting viral infections to serious bacterial infections (SBIs) including bacteraemia, urinary tract infection (UTI), pneumonia, bacterial meningitis, osteomyelitis, or septic arthritis. This systematic review was conducted to determine the thresholds at which C-reactive protein (CRP), procalcitonin (PCT), and interleukin-6 (IL-6) can predict SBIs in this population.

**Methods:**

We systematically searched electronic databases such as MEDLINE, Cochrane, CINAHL, and Web of Science for studies that evaluated the diagnostic accuracies of CRP, PCT, and IL-6 in detecting SBIs in children ≤36 months of age presenting with fever without source, during the period between November 2013 and November 2023. Area under the summary receiver operating curve (SROC) was calculated by the Rutter and Gatsonis method. *I*^2^ was used to quantify study heterogeneity. All tests were two-sided, and a *p*-value <0.05 was considered statistically significant. This review was registered with PROSPERO, CRD42023439093.

**Results:**

Datasets from 37 studies were included. A CRP cut-off of 10–20 mg/L had the highest pooled sensitivity of 0.75 (95% CI: 0.54–0.89), while a CRP cut-off of >40 mg/L had the highest pooled specificity 0.92 (95% CI: 0.87–0.95). A PCT cut-off of <0.5 ng/mL had the highest pooled sensitivity of 0.7812 (95% CI: 0.59–0.90) but the lowest pooled specificity of 0.69 (95% CI: 0.54–0.81). Based on receiver operating curve (ROC) analysis, a CRP cut-off between 10 and 20 mg/L and a PCT cut-off of <0.5 ng/mL showed the best diagnostic performance with a pooled AUC of 0.84 (95% CI: 0.79–0.90) and 0.816 (95% CI: 0.727–0.928), respectively. Only 1 study for IL-6 reported that a threshold of 20 pg/dL had a sensitivity and specificity of 79.1% and 91.6%, respectively.

**Conclusion:**

A PCT cut-off of 0.5 ng/mL and a CRP cut-off of 10–20 mg/L display the best performance in identifying SBIs in children ≤36 months of age with fever without source.

**Systematic Review Registration:**

https://www.crd.york.ac.uk/PROSPERO/view/CRD42023439093, identifier CRD42023439093.

## Introduction

Fever is the most common reason for paediatric emergency department visits (1). Approximately 20% of febrile young children have undifferentiated fever without an apparent source of infection (2). Although a majority of these patients have viral infections, a subset may have serious bacterial infections (SBIs) such as lobar pneumonia, urinary tract infections, bacteremia, and bacterial meningitis, which require appropriate management with antibiotics and hospitalization (3). While untreated SBIs can lead to significant morbidity and mortality, over-treatment of self-limiting viral illnesses can lead to antibiotic resistance and higher healthcare utilization and costs (4, 5).

Clinical evaluation is challenging in children younger than 36 months, as the features distinguishing self-limiting viral infections from SBIs are often subtle and can be easily missed. To address this, many studies have been performed to evaluate how accurately adjunct laboratory markers can help predict SBIs in this group of patients. White blood cell (WBC), C-reactive protein (CRP), and procalcitonin (PCT) have been widely reported as biomarkers of inflammation (6, 7). Indeed, these biomarkers are incorporated in clinical predictive algorithms to predict risks of SBIs among young children presenting with fever without source. More recently, newer evidence has highlighted the emerging role of cytokines such as interleukin-6 (IL-6) in predicting pediatric sepsis (8).

Interestingly, these biomarkers exhibit different kinetic profiles. CRP is an acute-phase reactant produced by the liver in response to tissue damage and infections, and its level can be elevated within 24 h of a trigger, peaks within 48 h, and has a half-life of 24 h (9–11). On the other hand, PCT levels can increase rapidly within 6–12 h in response to bacterial endotoxins, PCT peaks within 12 h, and it has a half-life of 24 h (12). As such, PCT has been regarded as a better biomarker to identify *early* bacterial infections compared with CRP (13). IL-6 is an inflammatory cytokine produced by monocytes and macrophages early in the inflammatory cascade, reaching its peak within 24 h but becoming undetectable within 24 h (14, 15).

Although the utility of these biomarkers has been widely reported and well established in the prediction of SBIs, the exact thresholds used to determine the likely presence of SBIs vary considerably among different studies (16–18). Therefore, in this study, we aimed to perform a systematic review and meta-analysis with the primary objective of determining the optimal thresholds at which CRP, PCT, and IL-6 can perform to distinguish the presence of SBIs in children younger than 36 months presenting with fever without source.

## Materials and methods

We adhered to the methods and procedures of the Preferred Reporting Items for Systematic Reviews and Meta-Analyses Diagnostic Test Accuracy (PRISMA-DTA) checklist for the reporting of this systematic review and meta-analysis (19). The study protocol has been published (20). This review was registered with the International Prospective Register of Systematic Reviews with registration number CRD42023439093.

### Eligibility criteria

We included studies conducted between November 2013 and November 2023 that reported specific thresholds and the corresponding diagnostic accuracies of CRP, PCT, and IL-6 in detecting SBIs in children younger than 36 months presenting with fever without source. Randomized controlled trials (RCTs) and non-randomized studies including non-RCTs, controlled before-and-after studies, and cohort studies were also included. We excluded case series, case-control studies, systematic reviews, meta-analyses, conference abstracts, and studies not reported in English. We also excluded studies that reported on both paediatric and adult patients but did not separately report on paediatric patients, studies with small population sizes (*N* < 50) (21), and studies that examined high-risk populations, specifically infants with prematurity and/or with a history of immunodeficiencies and malignancies.

### Information sources and search strategy

We performed a comprehensive search of the databases of MEDLINE, Cochrane Central Register of Controlled Trials, Cumulative Index to Nursing and Allied Health Literature (CINAHL), and Web of Science for pertinent studies. Additional references were identified by cross-checking bibliographies of full-text publications.

The search strategy was developed in collaboration with a librarian at our institution and is described in detail under Supplementary Information. The Medical Subject Headings (MeSH) terms “C-reactive protein,” “procalcitonin,” “interleukin-6,” “paediatrics,” “children,” “fever,” “pyrexia,” “infection,” and “sepsis” were used, and these terms were exploded where available. The search was conducted from 30 November 2023 to 1 December 2023. Duplicate results were removed.

### Study selection

Screening was conducted by five independent authors (NS, LJ, SY, SG, and CS-L) using Covidence, a software that follows the PRISMA 2009 guidelines that facilitate screening of studies during the selection process (22). Following initial screening of titles and abstracts, full-text articles of all relevant articles were retrieved and assessed based on study inclusion and exclusion criteria. Both stages of screening were performed by two independent reviewers, and any disagreements that followed were resolved by a third independent reviewer or by means of consensus.

### Data extraction and synthesis

All relevant data were extracted using standardized data collection forms on Microsoft Excel and verified. We extracted the following data from each included article: study title, authors, publication date and year, type of publication, country, study design, sample size, inclusion and exclusion criteria, clinical setting, characteristics of study population (including age, gender, and comorbidities), and duration of fever at the time of diagnostic workup. We documented the need for hospital admission and duration of hospitalization, as well as antibiotic use. We obtained the method of sampling CRP, PCT, and IL-6, values and units of measurement for CRP, PCT, and IL-6, and diagnostic performance indicators such as true positives (TPs), true negatives (TNs), false positives (FPs), and false negatives (FNs). However, a study was excluded when the aforementioned parameters were not mentioned or could not be obtained from the receiver operating curve (ROC). We also extracted unadjusted and adjusted odds ratios (ORs) and the unadjusted area under the curve (AUC) with a 95% confidence interval (CI).

### Definitions

1. Fever without source: temperature >38°C for less than 7 days without any signs or symptoms identifying the source of infection.
2. Early presentation: presentation of febrile children to the emergency department within 48 h of fever onset (23).
3. SBI: presence of bacteraemia, urinary tract infection (UTI), pneumonia, bacterial meningitis, osteomyelitis, or septic arthritis (24, 25).
4. Bacteraemia: growth of a single bacterial pathogen in blood, excluding growth of bacteria considered *a priori* as contaminants, for example, coagulase-negative *Staphylococcus* (26).
5. UTI: growth of single urine pathogen with at least 50,000 colony-forming units (CFUs)/mL from a catheterized urine specimen; or 10,000–50,000 CFUs/mL from a catheterized specimen with positive urinalysis (positive for leucocyte esterase, nitrite or pyuria at >5 white blood cells; or at least 100,000 CFUs/mL from urine collected via voided specimens) (27).
6. Pneumonia: lobar consolidation diagnosed on chest radiography confirmed by a paediatric radiologist (28).
7. Bacterial meningitis: cerebrospinal fluid leucocytes >5 cell/μL and positive bacterial culture (26, 29).
8. Osteomyelitis: positive bone scintigraphy.
9. Septic arthritis: positive bacterial culture of synovial fluid.

### Risk-of-bias assessment

We assessed the validity of each study using the Quality Assessment of Diagnostic Accuracy Studies (QUADAS-2) assessment tool, which provides a standardized way of assessing the risk of bias and applicability of diagnostic test accuracy research across four domains: patient selection, index test, reference standard, and flow and timing (30). Assessors are guided by signalling questions, which were adapted to this review, to reach a classification of “high,” “low,” or “unclear” for each subsection. The risk of bias or applicability was deemed to be “low risk” if all responses to signalling questions in that domain were affirmative; a classification of “high risk” was given if there were answers in the negative. If any question could not be answered because of a lack of information in the included study, the outcome for that subsection was “unclear.”

### Statistical analysis

The extracted number of TPs, FPs, FNs, and TNs from each study was pooled to calculate sensitivity, specificity, positive likelihood ratio, negative likelihood ratio, and diagnostic OR (DOR) using bivariate random-effects models for diagnostic meta-analysis. The summary receiver operating characteristic curve (SROC) was plotted, and the area under the SROC was calculated by the Rutter and Gatsonis method (hierarchical regression approach) (31). Based on available cut-offs from different studies, clinically meaningful categories were defined. For CRP, they were defined as <10, 10–<20, 20–<40, and ≥40 mg/L, while for procalcitonin, the cut-offs were <0.5 ng/mL, 0.5 ng/mL, and > 0.5–<2.0 mg/mL. A separate analysis was also done for CRP and procalcitonin to determine the best cut-off using Youden's index, with all the available cut-offs employed as continuous data.

Review Manager 5.4.1 (Cochrane Collaboration) analysis software was used to build the area under the SROC curve graphs by making use of different colours for different categories of CRP or procalcitonin cut-offs. Diagnostic power was considered good if the AUC was more than 0.8 and poor if it was less than 0.7. Publication bias was assessed using funnel plots, and Deek's asymmetry test was conducted to evaluate the publication bias for each category of CRP or procalcitonin cut-offs. A *p*-value of <0.10 indicated the presence of publication bias. All meta-analyses of unadjusted and adjusted ORs and the unadjusted AUC was performed using the DerSimonian Laird random effects model. Adjustments to ORs were based on the covariates identified in each individual study.

*I*^2^ statistic was used to quantify heterogeneity across studies, and an *I*^2^ statistic of 80% or more was considered to indicate considerable heterogeneity. However, owing to the broad review question resulting in an inherently heterogeneous population among included studies, we accepted *I*^2^ statistics up to 95% for inclusion in the meta-analysis (32). All tests were two–sided, and *p* < 0.05 was considered statistically significant. All analyses were carried out using Meta-DiSc 2.0 (Hospital Ramon y Cajal and Universidad Complutense de Madrid, Madrid, Spain), Revman 5.4.1, and R software version 4.2.2 (2022-10-31 ucrt).

## Results

**Figure 1:**
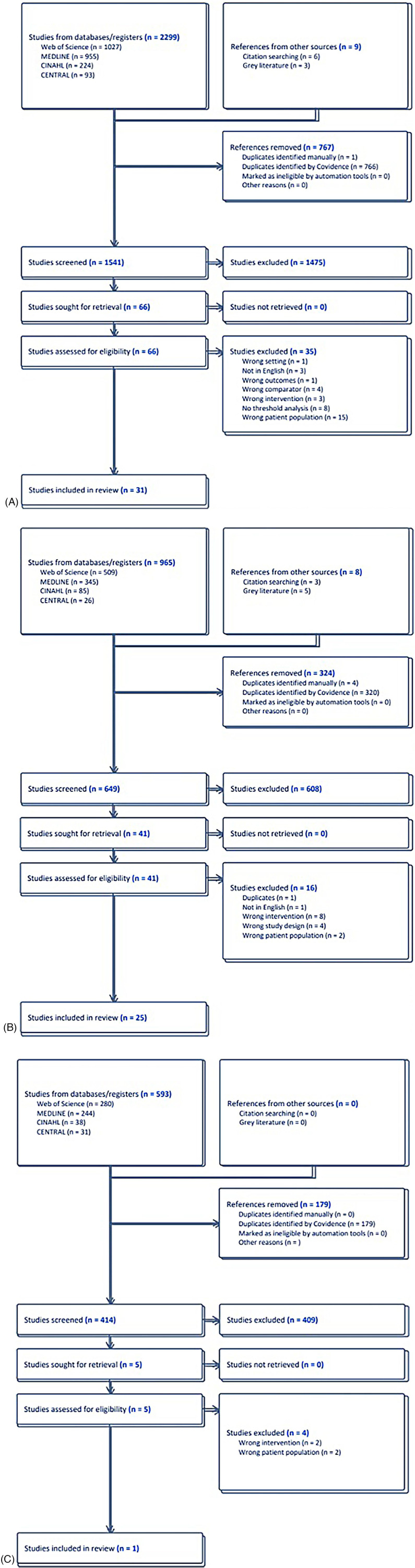
**(A)** Study selection for C-reactive protein. **(B)** Study selection for procalcitonin. **(C)** Study selection for interleukin-6.

Electronic database searches yielded a total of 2,299 publications with an additional 9 studies from manual searches for CRP, 965 publications with an additional 8 studies from manual searches for PCT, and 593 publications for IL-6 (Figures 1A–C). After exclusion of duplicates and screening of titles and abstracts of relevant publications, 66 studies were retrieved in full for review for CRP, 41 studies were retrieved in full for PCT, and 5 studies were retrieved for IL-6. Ultimately, datasets from 37 distinct studies were included in the meta-analysis, with 31 studies included in the meta-analysis for CRP and 25 studies included in the meta-analysis for PCT (Figures 1A–C). Only one study fulfilled the exclusion and inclusion criteria for IL-6.

The descriptive characteristics of the included studies, such as year of publication, study design and settings, number of eligible participants, and definitions of SBIs, are presented in Table 1. All studies were published in peer-reviewed journals. A majority (32 studies, 86.5%) of the studies were single-centre ones. Twenty-four studies (64.9%) had a retrospective study design. A majority (23 studies, 62.2%) included study participants up to 3 months of age. Fifteen studies (40.5%) defined SBIs as meningitis, bacteremia, UTI, and/or pneumonia, while eight studies (21.6%) defined SBIs as meningitis, bacteremia, and UTI. Seven studies (18.9%) included only the outcomes of bacteremia and/or meningitis. The reported rate of prevalence of SBIs ranged from 1.44% to 45.2%.

**Table 1:** Summary of studies included in the meta-analysis.

Citation	Country	Single or multi-centre	Study design	No. of participants	Age of study participants (months)	SBI prevalence (%)	Gender, female (%)	Cut-off values		Outcomes
								CRP (mg/L)	Procalcitonin (ng/mL)	
Sutiman et al. (33)	Singapore	Single	Prospective	258	0–3	86 (33.3)	109 (42.4)	20	0.5, 1.7	Meningitis, bacteremia, and UTI
Hiremath et al. (34)	India	Single	Explorative	31	3–36	14 (45.2)	12 (38.7)	10	0.5	Meningitis, bacteremia, UTI, and pneumonia
Nosrati et al. (35)	Israel	Single	Retrospective	401	0–3	48 (12.0)	177 (44.1)	2, 4, 5, 10, 20, 30, 40	N.A.	Meningitis, bacteremia, UTI, and pneumonia
Khoo et al. (36)	Singapore	Single	Retrospective	659	0–3	161 (24.4)	263 (39.9)	20	0.5	Meningitis, bacteremia, UTI, pneumonia, osteomyelitis, and septic arthritis
Manzano et al. (37)	Canada	Single	Prospective	328	1.36	54 (16.0)	163 (49.7)	17.7	0.2	Meningitis, bacteremia, UTI, pneumonia, osteomyelitis, and septic arthritis
Milcent et al. (38)	France	Multi	Prospective	2,047	0.23–3	139 (6.8)	829 (40.5)	20, 40	0.3, 0.5, 2	Meningitis, bacteremia, UTI, and pneumonia
Diaz et al. (39)	Spain	Single	Retrospective	318	0–3	76 (23.9)	128 (40.3)	3	0.5, 2	Meningitis and bacteremia
Lee et al. (40)	South Korea	Single	Retrospective	336	1–3	38 (11.3)	124 (36.9)	0.5	0.5	Meningitis, bacteremia, and UTI
Leroy et al. (41)	Switzerland	Multi	Retrospective	877	0–36	211 (24.1)	449 (51.2)	N.A.	0.5, 2	Meningitis, bacteremia, UTI, pneumonia, osteomyelitis, and septic arthritis
Chiu et al. (41)	Taiwan	Single	Retrospective	1,231	0–3	49 (3.98)	465 (37.8)	25	N.A.	Meningitis and bacteremia
D’Souza et al. (42)	India	Single	Retrospective	240	0–24	44 (18.3)	108 (45.0)	7	N.A.	Meningitis, bacteremia, UTI, and pneumonia
Galetto-Lacour et al. (43)	Switzerland	Single	Subpopulation of prospective database	135	0–36	20 (14.8)	55 (40.7)	40	0.5	Meningitis, bacteremia, UTI, pneumonia, and osteomyelitis
Hamiel et al. (44)	Israel	Single	Retrospective	1,039	0.23–3	111 (10.6)	411 (39.6)	5, 20, 40, 80	N.A.	Meningitis, bacteremia, and UTI
Gomez et al. (45)	Spain	Single	Subpopulation of prospective database	1,527	0–3	22 (1.44)	670 (43.9)	20	0.5	Meningitis, bacteremia, and UTI
Vujevic et al. (46)	Croatia	Single	Retrospective	181	0–3	70 (38.7)	71 (39.2)	13.3	N.A.	Meningitis, bacteremia, UTI, and pneumonia
Zarkesh et al. (47)	Iran	Single	Prospective	195	0–3	29 (14.9)	83 (42.6)	10	N.A.	Meningitis, bacteremia, UTI, and pneumonia
Polat et al. (48)	Turkey	Single	Retrospective	127	1–36	27 (21.3)	44 (34.6)	9	N.A.	Meningitis, bacteremia, UTI, and pneumonia
Rodriguez et al. (16)	Colombia	Single	Retrospective	137	0–36	40 (29.2)	78 (57.0)	80	N.A.	Meningitis, bacteremia, UTI, and pneumonia
Han et al. (49)	South Korea	Single	Retrospective	199	1–3	68 (34.2)	-	16.74, 20, 40	0.5, 0.6, 1.0	Meningitis, bacteremia, and UTI
Markic et al. (50)	Croatia	Single	Retrospective	135	0–6	62 (45.9)	64 (47.4)	40	0.5	Meningitis, bacteremia, UTI, and pneumonia
Moldovan et al. (51)	Romania	Single	Prospective	90	0.25–12	19 (21.1)	40 (44.4)	20, 40, 80	0.5, 2, 10	Meningitis, bacteremia, UTI, and pneumonia
Park et al. (52)	South Korea	Single	Retrospective	317	0–3	61 (19.2)	123 (38.8)	2	0.3	Meningitis, bacteremia, UTI, and pneumonia
Parada et al. (53)	Spain	Single	Retrospective	220	0–3	85 (38.6)	32 (40.0)	1, 2.75, 4, 10	0.26, 0.5, 1	Meningitis, bacteremia, UTI, and pneumonia
Li et al. (54)	Taiwan	Single	Retrospective	131	0–1	38 (29.0)	51 (39.0)	2.5	N.A.	Meningitis, bacteremia, UTI, and pneumonia
Hernandez-Bou et al. (55)	Spain	Single	Prospective	283	1–24	34 (12.0)	120 (42.4)	10	0.12	Meningitis and bacteremia
Kim et al. (56)	South Korea	Single	Retrospective	133	3–36	43 (32.0)	62 (46.6)	78	N.A.	Meningitis, bacteremia, UTI, pneumonia, osteomyelitis, and septic arthritis
Lacroix et al. (57)	Switzerland	Single	Prospective	241	0–36	41 (17.0)	93 (39.0)	40	0.5	Meningitis, bacteremia, UTI, pneumonia, osteomyelitis, and septic arthritis
Lejarzegi et al. (58)	Spain	Single	Prospective	2,850	0–3	534 (22.2)	1,207 (42.3)	60	20	Meningitis, bacteremia, and UTI
Lee et al. (59)	South Korea	Single	Retrospective	145	0–3	88 (60.7)	54 (37.2)	5, 10, 15, 20	N.A.	Meningitis only
Guo et al. (60)	China	Single	Retrospective	498	0–3	32 (6.4)	171 (34.3)	53.8	N.A.	Bacteremia only
Chang et al. (6)	Singapore	Single	Prospective	187	0–3	54 (28.9)	78 (41.7)	7.2	N.A.	Meningitis and bacteremia
Mahajan et al. (61)	USA	Multi	Prospective	226	0–36	30 (13.3)	131 (58.0)	N.A.	0.5	Meningitis, bacteremia, UTI, and pneumonia
Yin et al. (62)	China	Multi	Retrospective	1,053	0–0.93	166 (15.8)	432 (41.0)	N.A.	2.4, 18	Meningitis and bacteremia
Thongsamer et al. (63)	Thailand	Single	Retrospective	134	3–36	29 (21.6)	61 (45.5)	N.A.	0.5	Meningitis, bacteremia, UTI, and pneumonia
Kusma et al. (64)	USA	Single	Retrospective	663	0.27–2	121 (18.3)	287 (43.3)	N.A.	0.5	Meningitis, bacteremia, and UTI
Romain et al. (65)	France	Single	Retrospective	357	0.14–1	45 (12.6)	160 (44.8)	N.A.	0.6, 2	Meningitis, bacteremia, UTI, pneumonia, osteomyelitis, and septic arthritis
Kuppermann et al. (66)	USA	Multi	Prospective	1,821	0–2	88 (9.6)	765 (42.0)	N.A.	0.5, 1.7	Meningitis, bacteremia, and UTI

### Diagnostic performance of C-reactive protein in detecting SBIs

**Figure 2:**
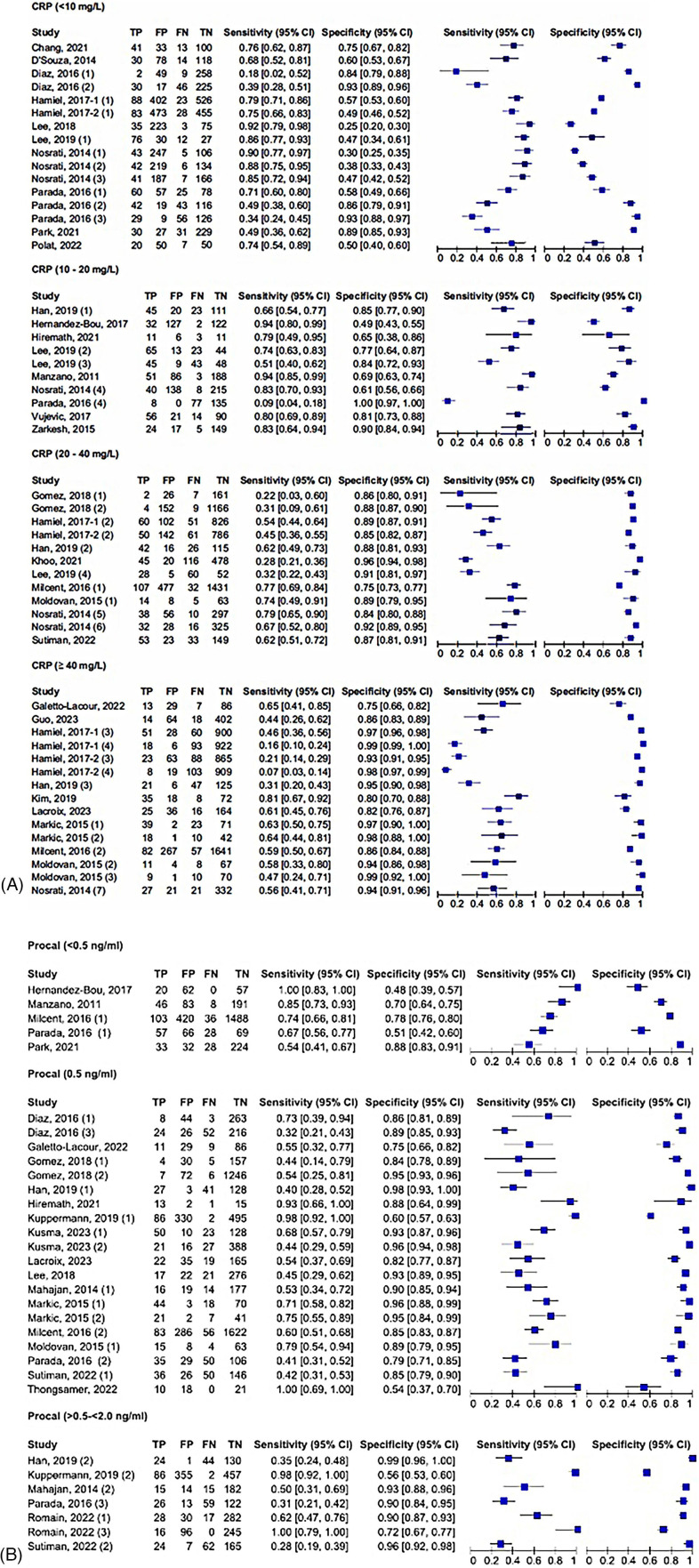
**(A)** Forest plots of sensitivity and specificity for different cut-offs of C-reactive protein for the detection of SBIs. **(B)** Forest plots of sensitivity and specificity for different cut-offs of procalcitonin for the detection of SBIs.

The unadjusted and adjusted odds ratios for the detection of SBIs were 9.37 (95% CI: 5.12–17.1, *I*^2^ = 95.9%) and 3.91 (95% CI: 2.13–7.16, *I*^2^ = 96.7%), respectively, for CRP (Tables 2, 3, respectively). The unadjusted AUC for CRP in detecting SBIs was 0.81 (95% CI: 0.78–0.85, *I*^2^ = 76.4%) (Table 4). For the detection of SBIs, CRP with a cut-off value between 10 and 20 mg/L had the highest pooled sensitivity of 0.75 (95% CI: 0.54–0.89) and DOR of 13.84 (95% CI: 8.84–21.70) (Figure 2A, Table 5). CRP with a cut-off value of more than 40 mg/L had the highest pooled specificity 0.92 (95% CI: 0.87–0.95) and positive and negative likelihood ratios of 6.31 (95% CI: 4.15–9.59) and 0.54 (95% CI: 0.43–0.69), respectively (Figure 2A, Table 5).

**Table 2:** Forest plot of unadjusted odds ratio (OR) for C-reactive protein in detecting SBIs.

Study	logOR	SE	Weight (common) (%)	Weight (random) (%)	Odds ratio IV, fixed + random, 95% CI	Odds ratio IV, fixed + random, 95% CI
Khoo, 2021	2.23	0.29	7.9	11.6	9.27 [5.27; 16.30]	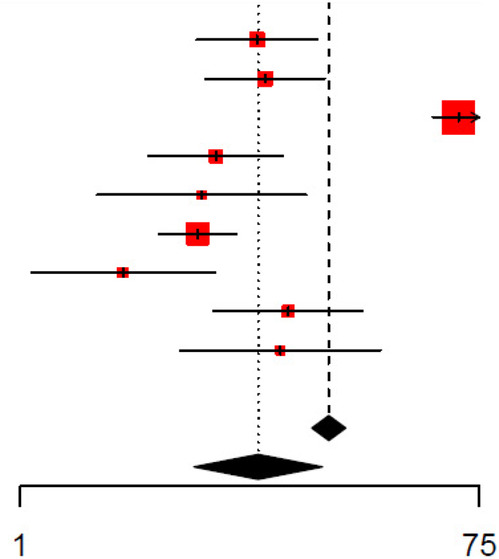
Milcent, 2016	2.30	0.29	8.0	11.6	10.00 [5.70; 17.54]	
Leroy, 2018	4.12	0.12	44.2	12.6	61.60 [48.50; 78.24]	
Chiu, 2018	1.84	0.32	6.3	11.3	6.30 [3.35; 11.85]	
Galetto−Lacour, 2022	1.71	0.50	2.6	9.6	5.51 [2.06; 14.74]	
Yin, 2022	1.67	0.18	19.6	12.3	5.30 [3.70; 7.59]	
Rodriguez, 2022	0.97	0.44	3.4	10.2	2.63 [1.11; 6.23]	
Han, 2019	2.52	0.36	5.1	11.0	12.42 [6.16; 25.04]	
Li, 2022	2.44	0.48	2.9	9.8	11.53 [4.51; 29.48]	
Total (common effect, 95% CI)			100.0		18.19 [15.52; 21.32]	
Total (random effect, 95% CI)				100.0	9.37 [5.12; 17.14]	
Heterogeneity: Tau^2^ = 0.7370; Chi^2^ = 192.85, df = 8 (*P* < 0.0001); *I*^2^ = 95.9% Test for overall effect (random effects): *Z* = 7.26 (*P* < 0.0001)						

**Table 3:** Forest plot of adjusted odds ratio (OR) for C-reactive protein in detecting SBIs.

Study	logOR	SE	Weight (common) (%)	Weight (random) (%)	Odds ratio IV, fixed + random, 95% CI	Odds ratio IV, fixed + random, 95% CI
Khoo, 2021	2.10	0.34	0.0	11.1	8.16 [4.15; 16.04]	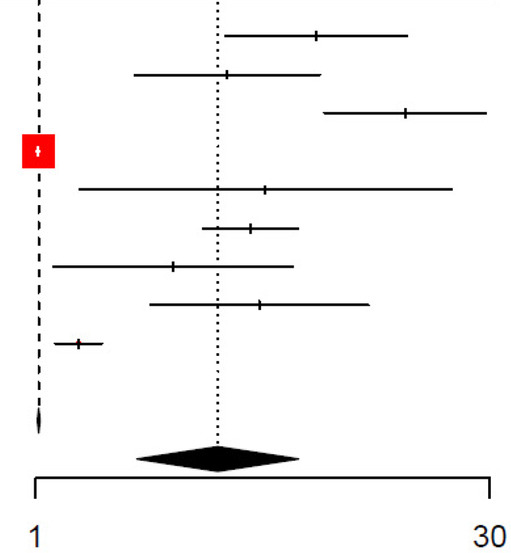
Milcent, 2016	1.44	0.35	0.0	11.1	4.20 [2.10; 8.40]	
Leroy, 2018	2.77	0.31	0.0	11.5	15.90 [8.70; 29.06]	
Chiu, 2018	0.02	0.00	99.5	12.9	1.02 [1.01; 1.03]	
Galetto−Lacour, 2022	1.72	0.71	0.0	7.7	5.59 [1.39; 22.48]	
Yin, 2022	1.61	0.18	0.1	12.4	5.00 [3.50; 7.14]	
Rodriguez, 2022	1.03	0.46	0.0	10.1	2.79 [1.14; 6.83]	
Han, 2019	1.67	0.42	0.0	10.5	5.33 [2.36; 12.04]	
Park, 2021	0.32	0.09	0.3	12.8	1.38 [1.16; 1.64]	
Total (common effect, 95% CI)			100.0		1.02 [1.01; 1.03]	
Total (random effect, 95% CI)				100.0	3.91 [2.13; 7.16]	
Heterogeneity: Tau^2^ = 0.7366; Chi^2^ = 245.70, df = 8 (*P* < 0.0001); *I*^2^ = 96.7% Test for overall effect (random effects): *Z* = 4.42 (*P* < 0.0001)						

**Table 4:** Forest plot of unadjusted area under the curve (AUC) for C-reactive protein in detecting SBIs.

Study	MD	SE	Weight (common) (%)	Weight (random) (%)	Mean difference IV, fixed + random, 95% CI	Mean difference IV, fixed + random, 95% CI
Sutiman, 2022	0.74	0.04	5.9	7.3	0.74 [0.67; 0.81]	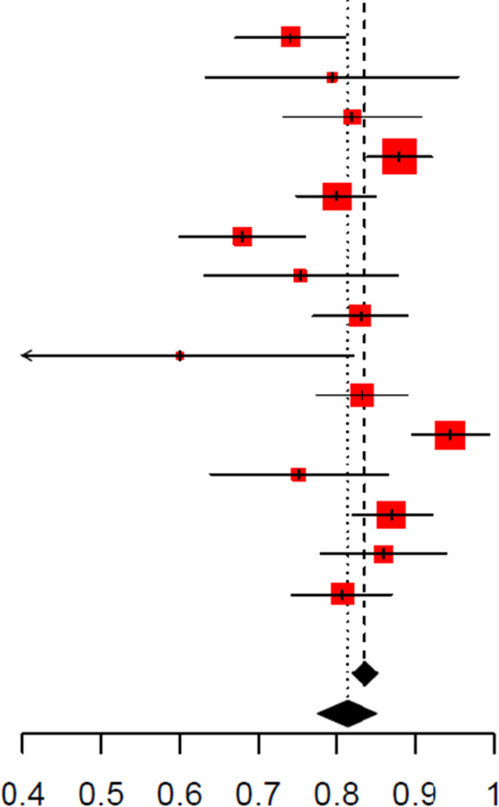
Hiremath, 2021	0.79	0.08	1.1	3.5	0.79 [0.63; 0.95]	
Nosrati, 2014	0.82	0.04	3.7	6.4	0.82 [0.73; 0.91]	
Manzano, 2011	0.88	0.02	17.7	8.8	0.88 [0.84; 0.92]	
Milcent, 2016	0.80	0.03	11.3	8.3	0.80 [0.75; 0.85]	
Diaz, 2016	0.68	0.04	4.4	6.8	0.68 [0.60; 0.76]	
Galetto−Lacour, 2022	0.75	0.06	1.9	4.8	0.75 [0.63; 0.88]	
Vujevic, 2017	0.83	0.03	7.9	7.8	0.83 [0.77; 0.89]	
Stein, 2014	0.60	0.11	0.6	2.3	0.60 [0.38; 0.82]	
Han, 2019	0.83	0.03	8.7	7.9	0.83 [0.77; 0.89]	
Markic, 2015	0.94	0.02	11.8	8.3	0.94 [0.90; 0.99]	
Parada, 2016	0.75	0.06	2.2	5.2	0.75 [0.64; 0.87]	
Hernandez−Bou, 2017	0.87	0.03	11.3	8.3	0.87 [0.82; 0.92]	
Kim, 2019	0.86	0.04	4.4	6.8	0.86 [0.78; 0.94]	
Lacroix, 2023	0.81	0.03	7.1	7.6	0.81 [0.74; 0.87]	
Total (common effect, 95% CI)			100.0		0.84 [0.82; 0.85]	
Total (random effect, 95% CI)				100.0	0.81 [0.78; 0.85]	
Heterogeneity: Tau^2^ = 0.0038; Chi^2^ = 59.21, df = 14 (*P* < 0.0001); *I*^2^ = 76.4% Test for overall effect (random effects): *Z* = 42.34 (*P* = 0)						

**Table 5:** Pooled diagnostic performance markers of C-reactive protein and procalcitonin for the detection of SBIs at multiple cut-offs.

Biomarker cut-offs	Sensitivity (95% CI)	Specificity (95% CI)	DOR (95% CI)	LR+ (95% CI)	LR− (95% CI)	AUC (95% CI)
C-reactive protein (mg/L)						
<10	0.71 (0.60–0.80)	0.64 (0.50–0.75)	4.34 (3.55–5.31)	1.96 (1.58–2.44)	0.45 (0.37–0.55)	0.79 (0.76–0.81)
10–20	0.75 (0.54–0.89)	0.82 (0.65–0.92)	13.8 (8.84–21.7)	4.20 (2.41–7.30)	0.30 (0.17–0.54)	0.84 (0.79–0.90)
20–40	0.54 (0.42–0.65)	0.88 (0.84–0.91)	8.66 (6.13–12.2)	4.54 (3.65–5.64)	0.52 (0.42–0.65)	0.78 (0.74–0.83)
>40	0.50 (0.37–0.63)	0.92 (0.87–0.95)	11.6 (7.089–19.1)	6.31 (4.15–9.59)	0.54 (0.43–0.69)	0.77 (0.72–0.84)
Procalcitonin (ng/mL)						
<0.5	0.78 (0.59–0.90)	0.69 (0.54–0.81)	8.02 (3.82–16.8)	2.54 (1.73–3.72)	0.32 (0.17–0.58)	0.82 (0.73–0.93)
0.5	0.64 (0.50–0.76)	0.87 (0.81–0.91)	12.1 (7.23–20.1)	4.95 (3.59–6.83)	0.41 (0.29–0.58)	0.81 (0.76–0.86)
>0.5–2.0	0.58 (0.29–0.82)	0.90 (0.79–0.96)	12.7 (6.75–24.0)	5.95 (3.71–9.56)	0.47 (0.25–0.88)	0.81 (0.74–0.90)

**Figure 3:**
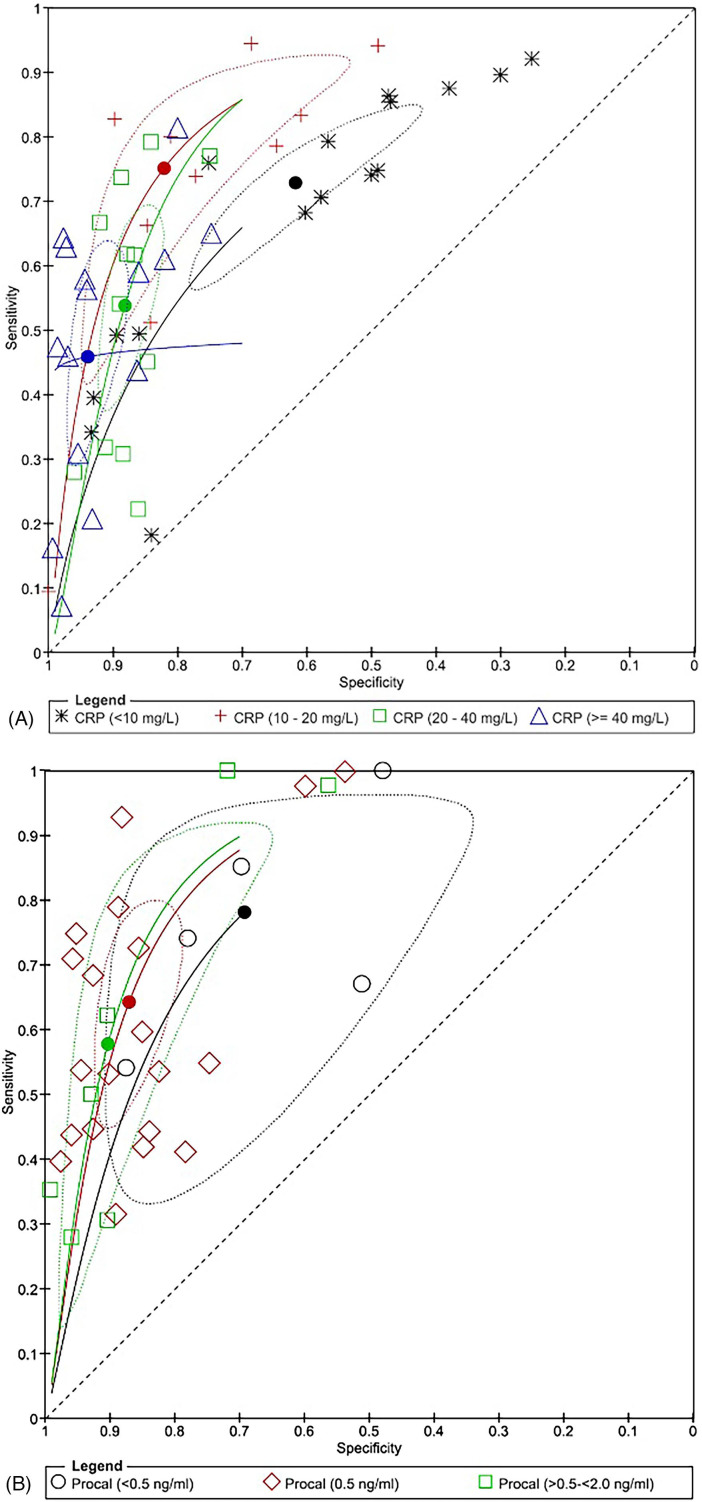
**(A)** Summary receiver operating characteristics (SROC) curve with confidence region for C-reactive protein to detect SBIs. **(B)** SROC curve with confidence region for procalcitonin to detect SBIs.

A CRP cut-off between 10 and 20 mg/L showed the best diagnostic performance with a pooled AUC of 0.84 (95% CI: 0.79–0.90). This was followed by an AUC of 0.79 for CRP <10 mg/L (95% CI: 0.76–0.81), 0.78 (95% CI: 0.74–0.83) for CRP between 20 and 40 mg/L, and 0.77 (95% CI: 0.72–0.84) for CRP more than 40 mg/L (Figure 3A, Table 5). Using a data-driven approach, the continuous threshold for CRP that showed the best diagnostic performance was 28.7 mg/L (Table 6). However, the sensitivity for a CRP cut-off of 28.7 mg/L was 0.55 (95% CI: 0.41–0.67) compared with the categorical threshold of 10–20 mg/L, which had a sensitivity of 0.75 (95% CI: 0.54–0.89).

**Table 6:** Best cut-off for C-reactive protein and procalcitonin as derived by Youden's index in detecting SBIs.

Biomarkers	Best cut-off	Sensitivity (95% CI)	Specificity (95% CI)
C-reactive protein (mg/L)	28.707	0.55 (0.41, 0.67)	0.86 (0.81, 0.90)
Procalcitonin (ng/mL)	0.039	0.66 (0.51, 0.78)	0.78 (0.70, 0.84)

Overall, a CRP threshold of 10–20 mg/L demonstrated the highest pooled sensitivity, whereas a CRP level above 40 mg/L offered the highest pooled specificity.

Further sub-analyses were conducted to explore the robustness of biomarker performance across clinically relevant strata, including age group (infants aged 0–90 days) and SBI prevalence (low- vs. high-prevalence cohorts using 10% and 20% cut-offs). In the 0–90-day subgroup, pooled estimates for CRP and PCT thresholds showed trends consistent with the main analysis; however, no statistically significant differences in sensitivity or specificity were observed. Similarly, stratification by SBI prevalence also did not reveal meaningful differences in diagnostic performance between low- and high-prevalence studies, and heterogeneity remained substantial across strata. Given the absence of significant effect modification and the limited number of studies contributing to some subgroups, these analyses were considered exploratory. Full results of these sub-analyses are reported as Supplementary Figure.

### Diagnostic performance of procalcitonin in detecting SBIs

For procalcitonin, the unadjusted and adjusted odds ratios were 4.62 (95% CI: 2.42–8.83, *I*^2^ = 97.3%) and 3.01 (95% CI: 1.60–5.65, *I*^2^ = 95.5%) (Tables 7 and 8, respectively), while the unadjusted AUC for procalcitonin was estimated to be 0.82 (95% CI: 0.73–0.93, *I*^2^ = 90.8%) (Table 9). Procalcitonin with a cut-off value of less than 0.5 ng/mL had the highest pooled sensitivity of 0.7812 (95% CI: 0.59–0.90) but the lowest pooled specificity of 0.69 (95% CI: 0.54–0.81) for the detection of SBIs (Figure 2B, Table 5). Procalcitonin between 0.5–2 ng/mL had the highest specificity at 0.90 (95% CI: 0.79–0.96) but the lowest sensitivity at 0.58 (95% CI: 0.29–0.82). At PCT between 0.5–2.0 ng/mL, the pooled DOR and positive and negative likelihood ratios were highest at 12.7 (95% CI: 6.75–24.0), 5.95 (95% CI: 3.71–9.56), and 0.47 (95% CI: 0.25–0.88), respectively (Figure 2B, Table 5).

**Table 7:** Forest plot of unadjusted odds ratio (OR) for Procalcitonin in detecting SBIs.

Study	logOR	SE	Weight (common) (%)	Weight (random) (%)	Odds ratio IV, fixed + random, 95% CI	Odds ratio IV, fixed + random, 95% CI
Khoo, 2021	1.04	0.49	0.8	11.1	2.84 [1.08; 7.47]	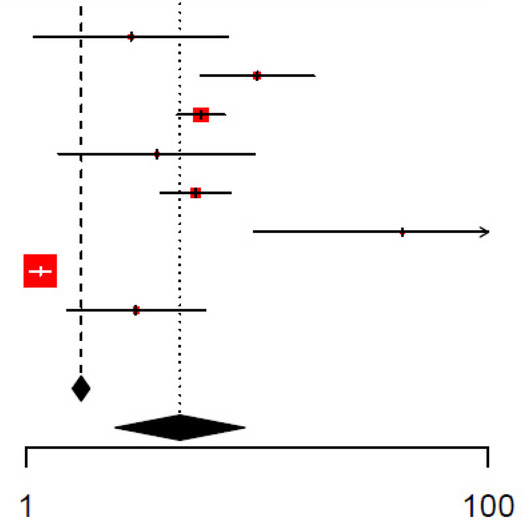
Milcent (1), 2016	2.30	0.29	2.4	13.3	10.00 [5.70; 17.54]	
Leroy, 2018	1.74	0.12	13.5	14.5	5.70 [4.50; 7.22]	
Galetto−Lacour, 2022	1.30	0.50	0.8	11.1	3.67 [1.38; 9.76]	
Yin, 2022	1.69	0.18	6.1	14.2	5.40 [3.80; 7.67]	
Han (3), 2019	3.75	0.75	0.3	8.4	42.48 [9.68; 186.42]	
Kusma (CVI−), 2023	0.14	0.05	74.5	14.8	1.15 [1.04; 1.27]	
Kusma (CVI+), 2023	1.09	0.35	1.6	12.7	2.98 [1.50; 5.92]	
Total (common effect, 95% CI)			100.0		1.73 [1.58; 1.88]	
Total (random effect, 95% CI)				100.0	4.62 [2.42; 8.83]	
Heterogeneity: Tau^2^ = 0.7368; Chi^2^ = 262.51, df = 7 (*P* < 0.0001); *I*^2^ = 97.3% Test for overall effect (random effects): *Z* = 4.63 (*P* < 0.0001)						

**Table 8:** Forest plot of adjusted odds ratio (OR) for Procalcitonin in detecting SBIs.

Study	logOR	SE	Weight (common) (%)	Weight (random) (%)	Odds ratio IV, fixed + random, 95% CI	Odds ratio IV, fixed + random, 95% CI
Khoo, 2021	0.21	0.60	0.0	10.1	1.23 [0.38; 3.98]	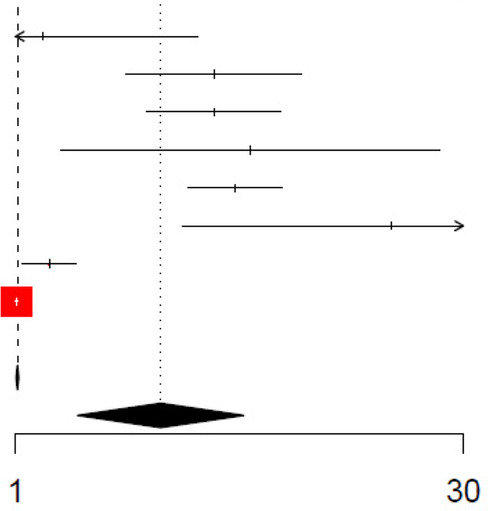
Milcent (1), 2016	1.50	0.34	0.0	13.3	4.50 [2.30; 8.80]	
Leroy, 2018	1.50	0.26	0.0	14.2	4.50 [2.70; 7.50]	
Galetto−Lacour, 2022	1.78	0.73	0.0	8.6	5.94 [1.41; 25.02]	
Yin, 2022	1.67	0.18	0.1	14.9	5.30 [3.70; 7.59]	
Han (3), 2019	2.85	0.81	0.0	7.9	17.33 [3.54; 84.84]	
Park, 2021	0.25	0.10	0.2	15.4	1.29 [1.05; 1.58]	
Lejarzegi, 2023	0.01	0.01	99.6	15.6	1.01 [1.00; 1.02]	
Total (common effect, 95% CI) total (random effect, 95% CI)			100.0	100.0	1.01 [1.00; 1.02]	
Total (random effect, 95% CI)				100.0	3.01 [1.60; 5.65]	
Heterogeneity: Tau^2^ = 0.6622; Chi^2^ = 156.94, df = 7 (*P* < 0.0001); *I*^2^ = 95.5% Test for overall effect (random effects): *Z* = 3.42 (*P* = 0.0006)						

**Table 9:** Forest plot of unadjusted area under the curve (AUC).

Study	MD	SE	Weight (common) (%)	Weight (random) (%)	Mean difference IV, fixed + random, 95% CI	Mean difference IV, fixed + random, 95% CI
Sutiman (1), 2022	0.63	0.04	3.4	5.8	0.63 [0.56; 0.71]	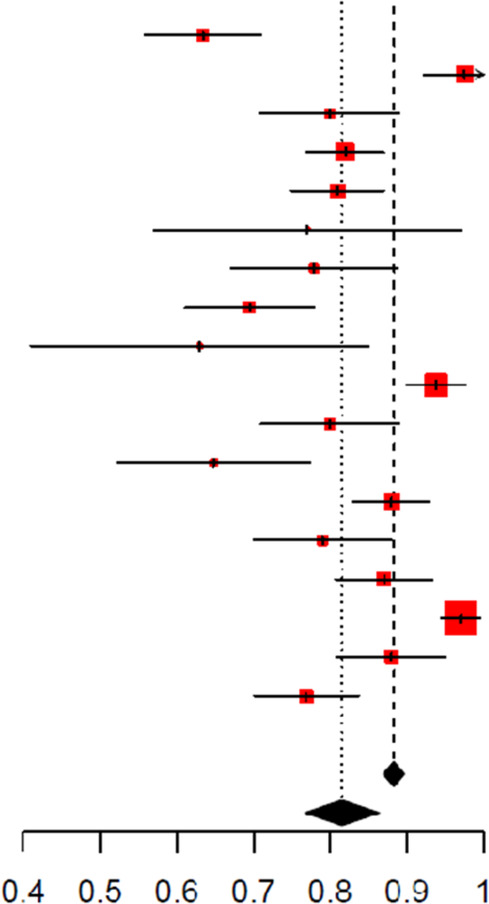
Hiremath, 2021	0.97	0.03	6.8	6.3	0.97 [0.92; 1.03]	
Mahajan (2), 2014	0.80	0.05	2.3	5.5	0.80 [0.71; 0.89]	
Manzano, 2011	0.82	0.03	7.6	6.3	0.82 [0.77; 0.87]	
Milcent (1), 2016	0.81	0.03	5.3	6.1	0.81 [0.75; 0.87]	
Diaz (IBI), 2016	0.77	0.10	0.5	3.2	0.77 [0.57; 0.97]	
Galetto−Lacour, 2022	0.78	0.06	1.6	5.1	0.78 [0.67; 0.89]	
Han (3), 2019	0.69	0.04	2.7	5.6	0.69 [0.61; 0.78]	
Stein, 2014	0.63	0.11	0.4	2.8	0.63 [0.41; 0.85]	
Markic (1), 2015	0.94	0.02	13.1	6.5	0.94 [0.90; 0.98]	
Thongsamer, 2022	0.80	0.05	2.3	5.5	0.80 [0.71; 0.89]	
Parada (1), 2016	0.65	0.06	1.2	4.7	0.65 [0.52; 0.77]	
Kusma (CVI−), 2023	0.88	0.03	7.6	6.3	0.88 [0.83; 0.93]	
Kusma (CVI+), 2023	0.79	0.05	2.3	5.5	0.79 [0.70; 0.88]	
Romain (SBI), 2022	0.87	0.03	4.8	6.1	0.87 [0.81; 0.93]	
Romain (IBI), 2022	0.97	0.01	30.1	6.7	0.97 [0.94; 1.00]	
Hernandez−Bou, 2017	0.88	0.04	3.9	5.9	0.88 [0.81; 0.95]	
Lacroix, 2023	0.77	0.03	4.1	6.0	0.77 [0.70; 0.84]	
Total (common effect, 95% CI)			100.0		0.88 [0.87; 0.90]	
Total (random effect, 95% CI)				100.0	0.82 [0.77; 0.86]	
Heterogeneity: Tau^2^ = 0.0090; Chi^2^ = 183.97, df = 17 (*P* < 0.0001); *I*^2^ = 90.8% Test for overall effect (random effects): *Z* = 32.97 (*P* < 0.0001)						

A PCT cut-off ≤0.5 ng/mL showed better performance based on SROC analysis (Figure 3B), although the pooled AUCs across the three cut-offs were similar (Table 5). Approaching procalcitonin as continuous data, the cut-off for procalcitonin that showed the best diagnostic performance was 0.039 ng/mL, which yielded a sensitivity of 0.66 (95% CI: 0.51–0.78) and a specificity of 0.78 (0.70–0.84).

Overall, PCT cut-off ≤0.5 ng/mL yielded the greatest pooled sensitivity but the lowest pooled specificity at 0.69 (95% CI: 0.54–0.81). Further sub-analysis could not be conducted for evaluation of PCT performance among different age groups and SBI prevalence in view of the small number of studies in each group.

### Diagnostic performance of IL-6 in detecting SBIs

Only one study ultimately met the inclusion and exclusion criteria for IL-6 and no meta-analysis was performed. Notably, Zarkesh et al. (47) reported that IL-6 at a threshold of 20 pg/dL had a sensitivity and specificity of 79.1% and 91.6%, respectively. In the same study, CRP at a threshold of 10 mg/L had a lower specificity of 78.2% but higher sensitivity at 81.6%. Both CRP and IL-6 were reported to be of higher predictive accuracy than WBC count and ANC in predicting SBIs.

### Risk-of-bias analysis

In this meta-analysis, Deeks' funnel plot asymmetry test was conducted to evaluate potential publication bias. No significant publication bias existed among the studies (*P*-value >0.2).

## Discussion

The inclusion of biomarkers in clinical decision support tools in the evaluation and management of febrile children presenting with fever without source has in large part been supported by the increasing evidence of their diagnostic performances in distinguishing SBIs from self-limiting viral illnesses (50, 67, 68). However, their optimal thresholds have remained inconclusive in view of high variability within and between studies. A recent systematic review and meta-analysis performed by Norman-Bruce et al. (69) evaluated the cut-offs of CRP and PCT for identification of invasive bacterial infections, but specifically only in febrile infants up to 90 days of life. With combined data from 37 unique data sets, this systematic review and meta-analysis is the latest and most comprehensive study to date to evaluate the thresholds at which the biomarkers CRP, PCT, and IL-6 can predict SBIs in children up to 36 months presenting with fever without source.

The results of this study suggest that CRP levels between 10 and 20 mg/L and PCT levels greater than 0.5 ng/mL have the best overall diagnostic accuracy in predicting SBIs in febrile children less than 36 months presenting with fever without source as reflected by their higher AUC values. These findings are in line with biomarker thresholds that have been validated in multiple clinical decision support tools such as Step-by-Step and AAP, both of which recommend cut-off values of 20 mg/L for CRP and 0.5 ng/mL for PCT (70, 71). We recognize that the high sensitivity of these thresholds is useful for ruling out SBIs, while moderate specificity may result in over-diagnosis and over-testing. Hence, these biomarkers have been incorporated in the algorithm in the aforementioned clinical decision support tools to limit unnecessary hospitalizations and invasive testing. Norman-Bruce et al. recently reported an optimum CRP level of 13.12 mg/L for the detection of invasive bacterial infections in febrile infants younger than 90 days and suggested the use of a lower cut-off of 15 mg/L.

The findings of this study also confirm that increasing the threshold value may increase specificity, enabling us to rule in the presence of SBIs with a greater degree of accuracy, with a CRP cut-off of 40 mg/L having the highest specificity of 0.92 (95% CI 0.87–0.95) and a PCT cut-off of above 0.5 ng/mL having the highest specificity of 0.90 (95% CI 0.79–0.96). Approaching CRP as a continuous variable, we determined the optimal threshold as 28.7 mg/L. However, this performed with a lower sensitivity compared with the categorical threshold of 10–20 mg/L. A higher threshold must be weighed against the risks of a higher false-negative rate, which may result in missed SBIs. Ultimately, the acceptable level of sensitivity and specificity and the decision on which threshold to use in clinical practice should also take into account heterogeneity of data and local prevalence of SBIs.

Indeed, there was high heterogeneity across studies, which may be due in part to differences in the definition of SBIs. The lack of a standardized definition of SBIs resulted in different infections with varying severity being identified across studies, which, in turn, led to variability in the outcomes assessed. This lack of standardization may result in either overestimation or underestimation of the association between biomarkers and SBI risk.

In addition, the majority of studies did not report the time at which the measurement of these biomarkers was performed. Given the pharmacokinetics of these biomarkers whereby CRP level reaches its maximum within 48 h, whereas PCT reaches its peak within 12 h (9, 10, 12, 13), this variability could introduce bias, particularly if some studies collect samples in the early stage of infection, while others do so in the later stage. Another factor contributing to heterogeneity is the age range differences between studies, particularly the distinction between neonates (0–3 months) and older infants (3–36 months). Younger children may present differently in terms of biomarker profiles and susceptibility to infections, influencing the biomarker thresholds used to identify SBIs. Furthermore, variability in laboratory methods, such as differences in assay techniques, sensitivity, and specificity, may lead to inconsistent results across studies. Finally, setting differences (e.g., emergency department vs. inpatient settings) may impact patient populations, with children in inpatient settings potentially reflecting more severe infections, thus affecting the generalizability of biomarker thresholds. Together, these factors contribute to heterogeneity by influencing both the detection of SBIs and the accuracy of biomarker thresholds, which may affect the overall precision of pooled estimates.

A majority of IL-6 studies were excluded primarily because they did not meet key eligibility criteria for this review, most commonly because of inappropriate age ranges, absence of confirmed SBI outcomes, or insufficient data to extract diagnostic accuracy measures or thresholds. As a result, only one study met the strict inclusion criteria, limiting the robustness of pooled estimates and preventing meaningful meta-analysis of IL-6 performance in this study. Although available data suggest potential diagnostic value, the lack of comparable studies precludes meaningful conclusions or recommendations for routine clinical use. Future research should prioritize standardization of IL-6 assays, including analytical methods, reference ranges, and reporting of thresholds. In addition, well-designed prospective studies should take into account the need to enrol a clearly defined population, apply consistent SBI definitions, report timing of biomarker sampling, and evaluate IL-6 as an independent marker alongside CRP and PCT.

This study has several strengths. First, we employed a comprehensive and rigorous search strategy, systematically screening four major databases with clearly defined inclusion and exclusion criteria. Our focus on young febrile children without a clear source of infection targets one of the most challenging clinical conundrums in evaluating febrile children up to 36 months of age. Second, we included only the most recent studies performed in the last 10 years to account for changing SBI epidemiology (72, 73). The inclusion of 37 studies in this study also allowed the aggregation of a large evidence base, hence strengthening the precision of pooled estimates. Methodologically, the use of hierarchical models, SROC curves, and diagnostic odds ratios ensured a robust synthesis of diagnostic performance of these biomarkers. In addition, the thresholds identified also showed clear alignment with existing clinical decision tools, including AAP and Step-by-Step approaches, supporting their applicability. We also provided transparent reporting of biomarker cut-offs and diagnostic accuracy metrics, with detailed tables and forest plots to facilitate interpretation by clinicians and researchers.

However, several limitations in this study must be acknowledged. Substantial heterogeneity across studies may weaken the reliability of pooled diagnostic estimates, due in part to the variability in SBI definitions and non-standardized timing of biomarker sampling, which represent a major potential source of bias. Evidence for IL-6 was extremely limited, with only one eligible study included. The dataset was also heavily weighted towards high-resource settings, which may limit generalizability to low- and middle-income contexts, and the exclusion of non-English publications may have introduced language bias. Finally, the predominance of retrospective study designs raises concerns about selection bias and incomplete ascertainment of SBI outcomes.

## Conclusions

The findings of this review suggest that PCT at a threshold of 0.5 ng/mL and CRP at a threshold of 10–20 mg/L show the best diagnostic performance in identifying SBIs in febrile young children less than 36 months of age presenting with fever without source. These findings need to be validated in a large multi-centre study using specific timings of biomarker measurements and outcome definitions, as well as calibration in the local context before implementation.

## Data Availability

The original contributions presented in the study are included in the article/Supplementary Material, and further inquiries can be directed to the corresponding author.
