# Supplementary material for: Identifying the thresholds of C-reactive protein, procalcitonin, and interleukin-6 among children ≤36 months’ old with fever without source at risk of serious bacterial infections: a systematic review and meta-analysis
Source: Front Pediatr. 2026 Feb 26;14:1697210. doi: 10.3389/fped.2026.1697210 (PMC12979460; doi:10.3389/fped.2026.1697210)
Supplement: Supplementary file 1 [file Supplementaryfile1.docx]

**Supplementary Information**

**Search strategy**

1. We considered each of the concepts and came up with a list of how they can be expressed. Based on our research question, the main concepts are : (i) fever, (ii) children aged 36 months or less, (iii) the different biomarkers, (iv) serious bacterial infections. We looked at alternative keywords and phrases for individual concepts and used MeSH on Pubmed.
2. We searched for initial results on Google Scholar and Pubmed and scanned these results for any other alternative words and phrases.
3. We looked at relevant abstracts from initial results and looked for alternative words or phrases that we may have missed out on.
4. We combined the search terms with "AND" or "OR" for the different concepts
5. At least 3 different databases were used in the search.

**Study period:** November 2013 to December 2023

**Key terms to search:**

(("febrile" OR "fever" OR "temperature" OR "pyrexia" OR "pyrexic")) AND (("C-reactive protein" OR "CRP")) AND (("Child" OR "children" OR "pediatric" OR "pediatrics" OR "paediatric" OR "paediatrics")) AND (("infection" OR "sepsis" OR "bacterial infection" OR “serious bacterial infection”))

(("febrile" OR "fever" OR "temperature" OR "pyrexia" OR "pyrexic")) AND (("procalcitonin" OR "procal" OR "pro-calcitonin" OR "PCT")) AND (("Child" OR "children" OR "pediatric" OR "pediatrics" OR "paediatric" OR "paediatrics")) AND (("infection" OR "sepsis" OR "bacterial infection" OR “serious bacterial infection”))

(("febrile" OR "fever" OR "temperature" OR "pyrexia" OR "pyrexic")) AND (("IL-6" OR "interleukin-6" OR "interleukin6")) AND (("Child" OR "children" OR "pediatric" OR "pediatrics" OR "paediatric" OR "paediatrics")) AND (("infection" OR "sepsis" OR "bacterial infection" OR “serious bacterial infection”))
