# Supplementary material for: Identifying the thresholds of C-reactive protein, procalcitonin, and interleukin-6 among children ≤36 months’ old with fever without source at risk of serious bacterial infections: a systematic review and meta-analysis
Source: Front Pediatr. 2026 Feb 26;14:1697210. doi: 10.3389/fped.2026.1697210 (PMC12979460; doi:10.3389/fped.2026.1697210)

Supplementary Figure 1B. Sub-group analysis for high disease prevalent studies (>10%)
Summary receiver operating charcteristic (SROC) curve with confidence region for CRP to detect SBIs

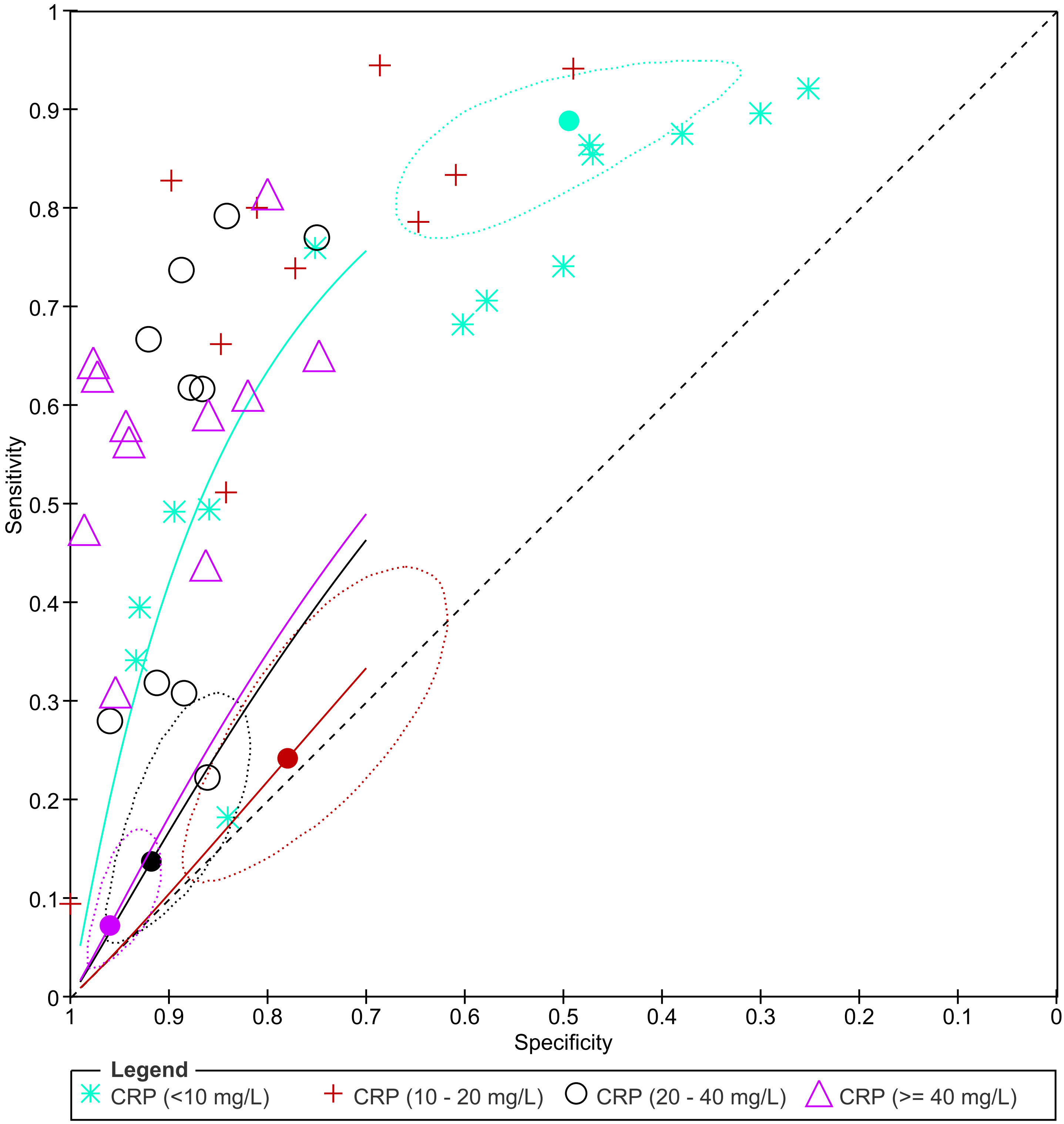

Supplement: Supplementary file 3 [file Datasheet2.pdf]
