# Supplementary material for: Identifying the thresholds of C-reactive protein, procalcitonin, and interleukin-6 among children ≤36 months’ old with fever without source at risk of serious bacterial infections: a systematic review and meta-analysis
Source: Front Pediatr. 2026 Feb 26;14:1697210. doi: 10.3389/fped.2026.1697210 (PMC12979460; doi:10.3389/fped.2026.1697210)

Supplementary Figure 2A. Sub-group analysis for low disease prevalent studies (≤20%)
Forest plots of sensitivity and specificity for different cut-offs of CRP for the detection of SBIs

CRP (<10 mg/L)

Study	TP	FP	FN	TN	Sensitivity (95% CI)	Specificity (95% CI)
D'Souza, 2014	30	78	14	118	0.68 [0.52, 0.81]	0.60 [0.53, 0.67]
Diaz, 2016 (1)	2	49	9	258	0.18 [0.02, 0.52]	0.84 [0.79, 0.88]
Lee, 2018	35	223	3	75	0.92 [0.79, 0.98]	0.25 [0.20, 0.30]
Nosrati, 2014 (1)	43	247	5	106	0.90 [0.77, 0.97]	0.30 [0.25, 0.35]
Nosrati, 2014 (2)	42	219	6	134	0.88 [0.75, 0.95]	0.38 [0.33, 0.43]
Nosrati, 2014 (3)	41	187	7	166	0.85 [0.72, 0.94]	0.47 [0.42, 0.52]
Park, 2021	30	27	31	229	0.49 [0.36, 0.62]	0.89 [0.85, 0.93]

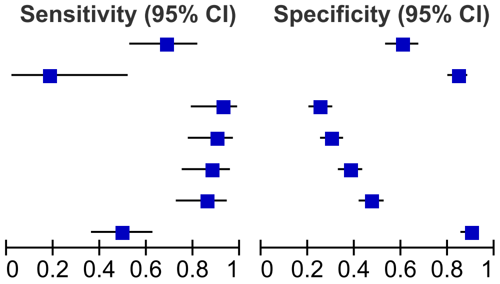

CRP (10 - 20 mg/L)

Study	TP	FP	FN	TN	Sensitivity (95% CI)	Specificity (95% CI)
Hernandez-Bou, 2017	32	127	2	122	0.94 [0.80, 0.99]	0.49 [0.43, 0.55]
Manzano, 2011	51	86	3	188	0.94 [0.85, 0.99]	0.69 [0.63, 0.74]
Nosrati, 2014 (4)	40	138	8	215	0.83 [0.70, 0.93]	0.61 [0.56, 0.66]
Zarkesh, 2015	24	17	5	149	0.83 [0.64, 0.94]	0.90 [0.84, 0.94]

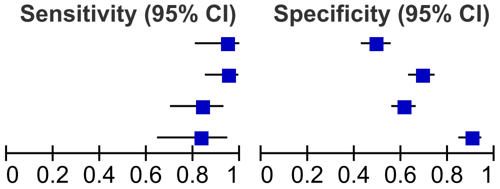

CRP (20 - 40 mg/L)

Study	TP	FP	FN	TN	Sensitivity (95% CI)	Specificity (95% CI)
Gomez, 2018 (1)	2	26	7	161	0.22 [0.03, 0.60]	0.86 [0.80, 0.91]
Gomez, 2018 (2)	4	152	9	1166	0.31 [0.09, 0.61]	0.88 [0.87, 0.90]
Milcent, 2016 (1)	107	477	32	1431	0.77 [0.69, 0.84]	0.75 [0.73, 0.77]
Nosrati, 2014 (5)	38	56	10	297	0.79 [0.65, 0.90]	0.84 [0.80, 0.88]
Nosrati, 2014 (6)	32	28	16	325	0.67 [0.52, 0.80]	0.92 [0.89, 0.95]

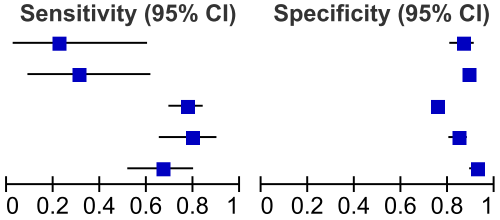

CRP (>= 40 mg/L)

Study	TP	FP	FN	TN	Sensitivity (95% CI)	Specificity (95% CI)
Galetto-Lacour, 2022	13	29	7	86	0.65 [0.41, 0.85]	0.75 [0.66, 0.82]
Guo, 2023	14	64	18	402	0.44 [0.26, 0.62]	0.86 [0.83, 0.89]
Lacroix, 2023	25	36	16	164	0.61 [0.45, 0.76]	0.82 [0.76, 0.87]
Milcent, 2016 (2)	82	267	57	1641	0.59 [0.50, 0.67]	0.86 [0.84, 0.88]
Nosrati, 2014 (7)	27	21	21	332	0.56 [0.41, 0.71]	0.94 [0.91, 0.96]

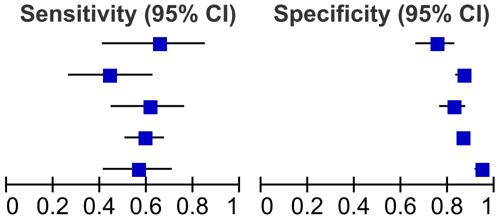

Supplement: Supplementary file 4 [file Datasheet3.pdf]
