# Supplementary material for: Identifying the thresholds of C-reactive protein, procalcitonin, and interleukin-6 among children ≤36 months’ old with fever without source at risk of serious bacterial infections: a systematic review and meta-analysis
Source: Front Pediatr. 2026 Feb 26;14:1697210. doi: 10.3389/fped.2026.1697210 (PMC12979460; doi:10.3389/fped.2026.1697210)

Supplementary Figure 4. Sub-analysis for 0-90 days
Forest plots of sensitivity and specificity for different cut-offs of CRP for detection of SBIs

CRP (<10 mg/L)

Study	TP	FP	FN	TN	Sensitivity (95% CI)	Specificity (95% CI)
Chang, 2021	41	33	13	100	0.76 [0.62, 0.87]	0.75 [0.67, 0.82]
D'Souza, 2014	30	78	14	118	0.68 [0.52, 0.81]	0.60 [0.53, 0.67]
Diaz, 2016 (1)	2	49	9	258	0.18 [0.02, 0.52]	0.84 [0.79, 0.88]
Diaz, 2016 (2)	30	17	46	225	0.39 [0.28, 0.51]	0.93 [0.89, 0.96]
Hamiel, 2017-1 (1)	88	402	23	526	0.79 [0.71, 0.86]	0.57 [0.53, 0.60]
Hamiel, 2017-2 (1)	83	473	28	455	0.75 [0.66, 0.83]	0.49 [0.46, 0.52]
Lee, 2018	35	223	3	75	0.92 [0.79, 0.98]	0.25 [0.20, 0.30]
Lee, 2019 (1)	76	30	12	27	0.86 [0.77, 0.93]	0.47 [0.34, 0.61]
Nosrati, 2014 (1)	43	247	5	106	0.90 [0.77, 0.97]	0.30 [0.25, 0.35]
Nosrati, 2014 (2)	42	219	6	134	0.88 [0.75, 0.95]	0.38 [0.33, 0.43]
Nosrati, 2014 (3)	41	187	7	166	0.85 [0.72, 0.94]	0.47 [0.42, 0.52]
Parada, 2016 (1)	60	57	25	78	0.71 [0.60, 0.80]	0.58 [0.49, 0.66]
Parada, 2016 (2)	42	19	43	116	0.49 [0.38, 0.60]	0.86 [0.79, 0.91]
Parada, 2016 (3)	29	9	56	126	0.34 [0.24, 0.45]	0.93 [0.88, 0.97]
Park, 2021	30	27	31	229	0.49 [0.36, 0.62]	0.89 [0.85, 0.93]

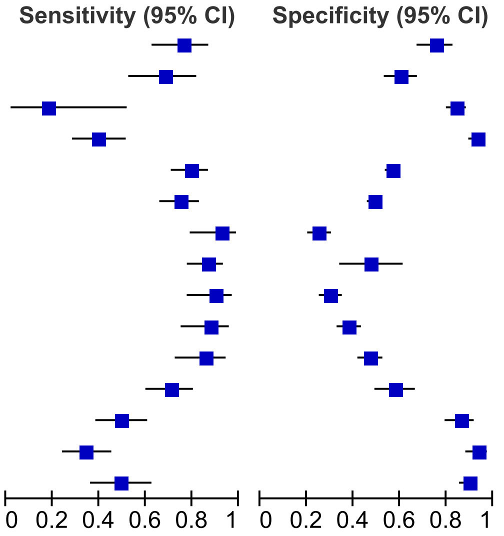

CRP (10 - 20 mg/L)

Study	TP	FP	FN	TN	Sensitivity (95% CI)	Specificity (95% CI)
Han, 2019 (1)	45	20	23	111	0.66 [0.54, 0.77]	0.85 [0.77, 0.90]
Lee, 2019 (2)	65	13	23	44	0.74 [0.63, 0.83]	0.77 [0.64, 0.87]
Lee, 2019 (3)	45	9	43	48	0.51 [0.40, 0.62]	0.84 [0.72, 0.93]
Nosrati, 2014 (4)	40	138	8	215	0.83 [0.70, 0.93]	0.61 [0.56, 0.66]
Parada, 2016 (4)	8	0	77	135	0.09 [0.04, 0.18]	1.00 [0.97, 1.00]
Vujevic, 2017	56	21	14	90	0.80 [0.69, 0.89]	0.81 [0.73, 0.88]
Zarkesh, 2015	24	17	5	149	0.83 [0.64, 0.94]	0.90 [0.84, 0.94]

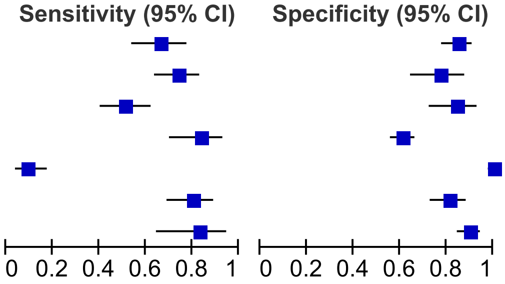

CRP (20 - 40 mg/L)

Study	TP	FP	FN	TN	Sensitivity (95% CI)	Specificity (95% CI)
Gomez, 2018 (1)	2	26	7	161	0.22 [0.03, 0.60]	0.86 [0.80, 0.91]
Gomez, 2018 (2)	4	152	9	1166	0.31 [0.09, 0.61]	0.88 [0.87, 0.90]
Hamiel, 2017-1 (2)	60	102	51	826	0.54 [0.44, 0.64]	0.89 [0.87, 0.91]
Hamiel, 2017-2 (2)	50	142	61	786	0.45 [0.36, 0.55]	0.85 [0.82, 0.87]
Han, 2019 (2)	42	16	26	115	0.62 [0.49, 0.73]	0.88 [0.81, 0.93]
Khoo, 2021	45	20	116	478	0.28 [0.21, 0.36]	0.96 [0.94, 0.98]
Lee, 2019 (4)	28	5	60	52	0.32 [0.22, 0.43]	0.91 [0.81, 0.97]
Milcent, 2016 (1)	107	477	32	1431	0.77 [0.69, 0.84]	0.75 [0.73, 0.77]
Nosrati, 2014 (5)	38	56	10	297	0.79 [0.65, 0.90]	0.84 [0.80, 0.88]
Nosrati, 2014 (6)	32	28	16	325	0.67 [0.52, 0.80]	0.92 [0.89, 0.95]
Sutiman, 2022	53	23	33	149	0.62 [0.51, 0.72]	0.87 [0.81, 0.91]

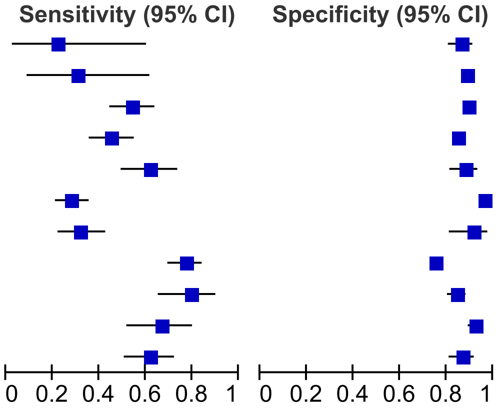

CRP (>= 40 mg/L)

Study	TP	FP	FN	TN	Sensitivity (95% CI)	Specificity (95% CI)
Guo, 2023	14	64	18	402	0.44 [0.26, 0.62]	0.86 [0.83, 0.89]
Hamiel, 2017-1 (3)	51	28	60	900	0.46 [0.36, 0.56]	0.97 [0.96, 0.98]
Hamiel, 2017-1 (4)	18	6	93	922	0.16 [0.10, 0.24]	0.99 [0.99, 1.00]
Hamiel, 2017-2 (3)	23	63	88	865	0.21 [0.14, 0.29]	0.93 [0.91, 0.95]
Hamiel, 2017-2 (4)	8	19	103	909	0.07 [0.03, 0.14]	0.98 [0.97, 0.99]
Han, 2019 (3)	21	6	47	125	0.31 [0.20, 0.43]	0.95 [0.90, 0.98]
Markic, 2015 (2)	18	1	10	42	0.64 [0.44, 0.81]	0.98 [0.88, 1.00]
Milcent, 2016 (2)	82	267	57	1641	0.59 [0.50, 0.67]	0.86 [0.84, 0.88]
Nosrati, 2014 (7)	27	21	21	332	0.56 [0.41, 0.71]	0.94 [0.91, 0.96]

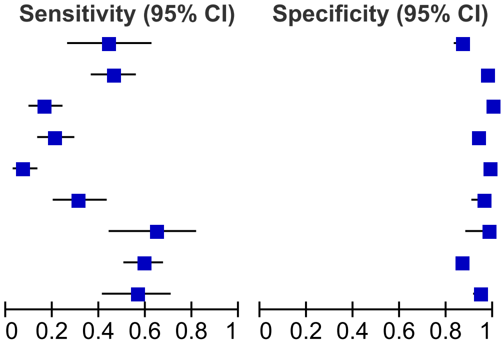

Supplement: Supplementary file 8 [file Datasheet7.pdf]
